# Crop Resilience to Drought With and Without Response Diversity

**DOI:** 10.3389/fpls.2020.00721

**Published:** 2020-06-03

**Authors:** Heba H. Elsalahy, Sonoko D. Bellingrath-Kimura, Christina-Luise Roß, Timo Kautz, Thomas F. Döring

**Affiliations:** ^1^Albrecht Daniel Thaer-Institute of Agricultural and Horticultural Sciences – Crop Science, Humboldt-University of Berlin, Berlin, Germany; ^2^Botany and Microbiology Department, Faculty of Science, Assiut University, Assiut, Egypt; ^3^Research Area “Land Use and Governance”, Leibniz Centre for Agricultural Landscape Research (ZALF), Müncheberg, Germany; ^4^Agroecology and Organic Farming Group, University of Bonn, Bonn, Germany

**Keywords:** agroecosystems, aridity, binary mixture, drought resistance, forage legumes, functional traits, monoculture

## Abstract

In the face of increasingly frequent droughts threatening crop performance, ecological theory suggests that higher species diversity may help buffering productivity by making systems more resistant through resource complementarity and more resilient through higher response diversity. However, empirical evidence for these diversity effects under drought stress has remained patchy. In two pot experiments, we explored whether mixing two legume species with a contrasting response to water availability, alsike clover (AC) and black medic (BM), promotes resistance to cumulative drought stress, and resilience of aboveground crop biomass to a transient drought event. The mixture was more productive than the average of the sole crops, and this mixture effect was higher in the non-stressed than in the drought-stressed plants. However, with six levels of constant drought intensities, the mixture effect was not consistently affected by drought level. Response diversity was evident as asynchrony of growth in the two species after the drought event, with BM re-growing faster than AC. Significant resilience to drought was observed in sole AC, i.e., without response diversity. Resilience was larger in AC than in BM and increased from 44 to 72 days after sowing (DAS). The mixture was more resilient than the average resilience of the sole crops at 72 DAS, but it was never more resilient than AC, indicating that resilience is promoted by, but not dependent on response diversity. We conclude that crop diversity may contribute to drought resilience through growth asynchrony, but that species identity plays a crucial role in making systems more drought-resilient.

## Introduction

Climate change is projected to show increasing frequency and intensity of ecosystem disturbances such as irregular rainfall patterns, including extreme drought events (IPCC, 2013; Huang et al., 2016). Drought can affect crop yield directly or through complex interactions with soil properties, nutrient availability, and temperature stress (Mariotte et al., 2018) or with soil biota such as mycorrhiza (Schimel, 2018). Two scenarios of drought may affect crop production, cumulative drought stress (Swemmer et al., 2007; Navarro-Cerrillo et al., 2018), and periodic (transient) drought stress (Hofer et al., 2016; Komainda et al., 2019). While plants can withstand moderate changes in total annual precipitation, increased variability in the amount of precipitation per event and in the event duration can substantially impair aboveground biomass production (Swemmer et al., 2007). Therefore, strategies to cope with drought in natural and managed ecosystems need to be found to maintain overall ecosystem stability, either through drought resistance, or through drought resilience, i.e., the ability to recover from drought events (Hoover et al., 2014; Hodgson et al., 2015).

Ecological theory suggests that both resistance and resilience to stress may be promoted by diversity. The underlying mechanisms behind benefits of diversity for resistance and resilience are related to the insurance hypothesis (Yachi and Loreau, 1999; Morin et al., 2014), which predicts that ecosystems with high diversity provide a buffer against environmental fluctuations (Ouédraogo et al., 2013; Oliver et al., 2015). In particular, increased plant species richness may enhance resistance through complementarity of resource use and concurrently lower niche overlap, e.g., with spatially or temporally different rooting patterns of the component species. Resilience, on the other hand, may also be enhanced by diversity. Specifically, it has been suggested that resilience critically depends on response diversity (Elmqvist et al., 2003; Mori et al., 2013). Combining species that are similar in their ability to fulfill a given function, but diverse in their responses to a particular stress may enhance the resilience of the community performance with respect to that function. Diversity within functional groups (i.e., ecological redundancy) may be critical for recovering ecosystem services after stress, as response diversity creates temporal niche differentiation allowing for compensatory dynamics among species (Loreau and de Mazancourt, 2008; Yu et al., 2016). The stress event may lead to a re-ordering of species dominance within a community, with species less affected by the stress becoming dominant because competition from the stress-affected species is relaxed (Hoover et al., 2014).

So far, however, findings have been mixed for the effect of plant diversity on both drought resistance and drought resilience. For example, while a seminal study in grassland showed that species richness promotes drought resistance (Tilman and Downing, 1994), there have also been results showing that drought resistance is not always enhanced by plant diversity, e.g., in grassland (Van Ruijven and Berendse, 2010; Lanta et al., 2012) and forest ecosystems (Grossiord et al., 2014). Also, while some studies support the diversity-resilience hypothesis (Van Peer et al., 2001; Allsion, 2004; Di Falco and Chavas, 2008; Hutchison et al., 2018), there was no positive association between response diversity and resilience in other studies (Barkaoui et al., 2016; Fischer et al., 2016; Bhaskar et al., 2018). Further, research on the diversity-resilience relationship has so far mostly focused on permanent grasslands or forests, concentrating on longer-term, i.e., multiannual, effects through shifts in species composition (Tilman and Downing, 1994; Ouédraogo et al., 2013; Fischer et al., 2016; Hofer et al., 2016). In contrast, evidence for effects of response diversity on resilience remains scarce for arable cropping systems, where shorter-term, i.e., within-season effects are more relevant than year-to-year resilience, and where planned species diversity is most often much lower than in grasslands.

Therefore, we studied the effect of plant diversity on drought resistance and resilience in an arable cropping context, using the simplest possible design, by testing sole crops of two different plant species against their binary mixture. We chose two legume species, alsike clover (AC; *Trifolium hybridum* L.) and black medic (BM; *Medicago lupulina* L.), thus both belong to the same functional group. We only varied species richness and no other parameter of diversity to ensure that differences in response diversity were as large as possible. Legumes play a vital role in arable cropping systems because of their ability to fix atmospheric nitrogen, but also because they increase soil organic matter levels, facilitate soil nutrient circulation and suppress weeds (Döring et al., 2013; Stagnari et al., 2017). While both species are also found in permanent grassland, they are frequently used as short-term green manures in arable systems to increase soil fertility, to attract beneficial insects or as living mulches to suppress weeds (Döring et al., 2013; Elsalahy et al., 2019). The selected species are characterized by various contrasting traits. BM is a fast-growing perennial well-adapted to warm and dry areas (Chapman et al., 1990; Döring et al., 2013; Elsalahy et al., 2019; FAO, 2019); it has a short-medium and spreading growth habit forming good ground cover (Floulds, 1978). Conversely, AC is a slow-growing drought-sensitive perennial, best adapted to cool and wet areas (Sheaffer et al., 2003; Döring et al., 2013) which has an upright growth habit with a single crown from which multiple florets are produced (Frankow-Lindberg et al., 2009; Storkey et al., 2015).

We used two pot experiments to quantify resistance and resilience of the two legumes, sown as sole crops and in an equiproportional mixture. Specifically, we aimed to test if the mixture shows higher resilience and resistance to drought stress than the two sole crops. Drought resistance was quantified in response to cumulative drought (CD) with six levels of drought intensities whereas resilience was quantified by comparing plants exposed to a single transient drought event (DE) to non-stressed plants. Our key hypotheses were: (1) The two species differ in their drought resistance and resilience. (2) Because of resource complementarity, the mixture is more drought-resistant than the sole crops. (3) Because of response diversity, the mixture is more drought-resilient than the sole crops.

## Materials and Methods

### General Set-Up

Two pot experiments were conducted at the Humboldt University experimental station at Berlin-Dahlem in a greenhouse protected on all sides with a wire mesh (25.4 × 25.4 mm clear opening, 3.18 mm Ø wire). The mesh size allowed insects, wind, and temperature to be inside the greenhouse as in the surrounding field but prevented any larger animals from entering. The greenhouse roof was covered with polycarbonate plastic panels (16 mm triple wall) transmitting 76% of ambient light.

In both experiments, three mixture treatments were used, a sole crop of AC (cv. Dawn), a sole crop of BM (cv. Ekola), and a 1:1 mixture. The seeds were bought from Deutsche Saatveredelung AG (DSV) and Camena Samen, Germany for AC and BM, respectively. The selection of 1:1 mixing ratio is based on two field experiments conducted over 2 years, where we tested the two species at five mixing ratios of AC:BM (100:0, 67:33, 50:50, 33:67, and 0:100) sown at three seed densities representing 50, 100, and 150% of the recommended seed density. The results showed that the equiproportional mixture of the two species had a stronger (or more consistent) positive mixture effect in comparison with the other mixing ratios (Elsalahy et al., 2019). Seed density was 24 seeds per pot, with seeds of the two species being spatially alternated in the mixture to ensure maximal interspecific interaction. Any emerged weed seedlings were removed daily from the pots. Precise irrigation was facilitated by using a dispenser (Rotilabo^®^-Dispenser 20–100 ml, accuracy 1 ml, by Roth, Germany).

In both experiments, the plants were sampled by cutting them ca. 0.5 cm above the soil surface. In the mixture, the harvested material of the two species was manually separated and bagged separately into transparent microperforated plastic bags, made of SM570Y film (Cryovac^®^, Sealed Air Corporation, Elmwood Park, NJ, United States) and oven-dried (Thermo Scientific Heraeus UT 6760, Germany) at 65°C for 72 h to obtain constant weight.

### Drought Resistance Experiment

The experiment was started on 1st June 2016 and run for 50 days to simulate the effect of cumulative drought. Six drought intensities were established with varied levels of water holding capacity (WHC) of the soil. The average daily temperature during the period of the experiment was 18.2°C, ranging from 15.1 to 27.2°C. The average daily radiation was 20.0 MJ m^–2^ d^–1^, ranging from 6.1 to 30.4 (Agricultural Climatology of the Hu-Berlin, 2018).

One day before sowing, square pots (12 × 12 × 19 cm) were filled with 3430 g substrate collected from the top soil (upper ∼15 cm) of a non-cropped bare field at the experimental station of Humboldt University of Berlin in Dahlem (52° 28′ N, 13° 18′ E, 51 m asl). The soil, a sandy clay loam (Brady and Weil, 2002), had a pH of 6.3, organic matter content of 1.24%, nitrogen content of 0.13%, and nutrient contents per kg soil of 251 mg P, 90 mg K, 52 mg Mg, 1471 mg Ca, and 7354 mg Fe^3+^. Soil WHC was measured in three replicates and calculated following Nguyen and Lehmann (2009).

The experimental design was a two-factorial randomized complete block design in four replicates with the factor diversity (called *DIV*) comprising three diversity treatments (two monocultures and one mixture), and the drought factor (called *CD*), with six levels of cumulative drought intensity (see below). Each block contained 18 pots and one control pot without plants to calculate the daily evaporated water (Supplementary Figure S2).

On the day of sowing, 478 ml distilled were added to each pot to moisten the soil and to facilitate placing the seeds in fixed distances without silting up the soil surface. Seeds were sown at a depth of 0.5 cm with a handmade wooden seed stamp in a design of 6 × 4 to allow 2 cm space between sown seeds. Three days later, an equal amount of water was added to all treatments to compensate for evaporation and ensure optimum conditions for germination in all treatments by keeping WHC at ∼90% before starting the different irrigation regimes.

One week after sowing, differentiated irrigation was initiated, creating six different levels. The average weight of the control pots (without plants) was determined to calculate the amount of evaporated water. This amount was then given to the 100% WHC level and reduced to 85, 70, 55, 40, and 25% of WHC (Supplementary Figure S1A). Three weeks later, an additional amount of water was added to compensate for transpiration. To keep the relative differences fixed between the treatments, the added amounts to compensate transpiration was summed to the evaporated water and reduced gradually according to the previously determined levels. E.g., when the pots were irrigated with 200 ml for evaporation and 100 ml for transpiration, the sum of 300 ml represented 100% WHC and was reduced accordingly for the other levels. Cover crop aboveground biomass (CCB) was harvested once at the end of the experiment.

### Drought Resilience Experiment

The experiment was started on 26th June 2017 and was run for 72 days. The average daily temperature during the period of the experiment was 18.2°C, ranging from 13.5 to 24.6°C and the average daily radiation was 13.5 GS MJ m^–2^ d^–1^, ranging from 1.2 to 24.5 (Agricultural Climatology of the Hu-Berlin, 2018).

Round pots (3.5 L, Ø12 × 25 cm height) were filled with soil collected from the top-soil (upper ∼15 cm) of a non-cropped bare field at the experimental field station of the Humboldt University of Berlin in Dahlem. The soil was sandy loam (Brady and Weil, 2002) with a pH-value of 6.3, organic matter content of 0.72%, nitrogen content of 0.09%, and nutrient contents per kg soil of 121 mg P, 83 mg K, 37 mg Mg, 1242 mg Ca, and 5044 mg Fe^3+^. The pots were filled with the soil and covered with a circular filter paper and 500 ml of tap water was poured carefully onto the filter paper. Soil WHC was measured as reported above.

The experimental design was a three-factorial randomized complete block design in five replicates with three diversity treatments (called *DIV*; two monocultures and one mixture), two drought event treatments (called *DE*, non-stressed and drought-stressed), and five prospective harvest times (called *Har*, H1 to H5). Each block contained 30 pots and one control pot without plants to determine daily evaporated water (Supplementary Figure S3).

One day before sowing, the pots were filled with 3392 g of soil. On the next day, the soil was moistened with 25 ml water after placing a filter paper on the soil surface to avoid silting up. Then, the seeds were manually placed on top of the wet substrate in a regular pattern, using a sowing stencil to mark the seeds’ positions. Immediately after placing the seeds, 108 g of loosely dry soil was spread evenly on top of the soil surface to cover the sown seeds. The filter paper was again placed on the top of the soil surface while adding 500 ml of water to adjust WHC at ∼90%. Afterward, until 22 DAS, all non-stressed plants and stressed plants were irrigated equally and received an amount of cumulative applied water equal to 0.98 L pot^–1^ (Supplementary Figure S1B).

At 23 DAS (H1), a drought event was started for 14 days. During the drought event, the stressed plants received 33% of the water added to the non-stressed plants. At 37 DAS (H2), the drought stress was released by re-watering the non-stressed plants and stressed plants daily with the same amounts of water. Plant recovery, namely plant growth after the drought event was evaluated at 44, 58, and 72 DAS. At each of the five harvest times, different pots were harvested to evaluate the temporal development of CCB production over the course of the experiment.

### Calculation of Indices

In both experiments, water use efficiency (WUE, in g DM *L*^–1^) was determined by using Equation (1) (Komainda et al., 2019).

(1) $$\text{WUE}=b_{i}/w_{i}$$

where *b*_i_ is CCB in g per pot, produced at harvest i, and *w*_i_ is the irrigation water in *L* per pot cumulated until the harvest i.

In the resilience experiment, crop growth rate (CGR in g m^–2^ d^–1^), defined as the CCB per unit ground area per unit time (g m^–2^ day^–1^) was calculated by using Equation (2) (Poorter, 1989).

(2) $${\text{CGR}}_{\text{i}}=\left(b_{i}-b_{i-1}\right)/\left[A\,\left(t_{i}-t_{i-1}\right)\right]$$

where *b*_i_ and *b*_i–1_ are dry matter in g per pot at the end and beginning of a time interval, respectively, i.e., at harvest i and i-1, thus, *t*_i_–*t*_i–1_ is the time interval in days between two consecutive harvest times; and *A* is the ground area of the pot in m^2^.

To quantify the effect of the mixture in comparison to the monocultures, land equivalent ratio (LER) and partial LER (PLER) of the two species were calculated for the CCB (g DM pot^–1^). The LER of a mixture measures the relative land area that is required for the crop monocultures to produce the same CCB as observed in the mixture; it was calculated as the sum of the PLERs of the two species in the mixture by using Equation (3) (Mead and Willey, 1980).

(3) $$\text{LER}={\text{PLER}}_{\text{AC}}+{\text{PLER}}_{\text{BM}}=\frac{b_{\mathrm{AC}\,\_\,\mathrm{mix}}}{b_{\mathrm{AC}\,\_\,\mathrm{mono}}}+\frac{b_{\mathrm{BM}\,\_\,\mathrm{mix}}}{b_{\text{BM\_mono}}}$$

where b_AC_mono_ and b_BM_mono_ are the biomass of species AC and BM in monoculture and b_AC_mix_ and b_BM_mix_ are the biomass of each species in the mixture. An LER >1 indicates that the mixture makes more efficient use of the land and has an advantage over the monoculture. Partial LERs show the relative competitive abilities of each species in the mixture and can be interpreted as a measure for the contribution of each species according to its density ratio in the mixture relative to the monoculture.

For CCB, WUE, and CGR, the absolute mixture effect (AME) was calculated as the difference between the observed value in the mixture and the average values of the two monocultures (Equation 4). For the same variables, the relative change in response to stress (Hofer et al., 2016) was calculated by using Equation (5).

(4) $$\text{AME}=y_{m\,i\,x}-\left(y_{A\,C}+y_{B\,M}\right)/2$$

(5) $$Changeinvariabley\left(\%\right)=100^{*}\left(y_{\mathrm{stressedplants}}/y_{\mathrm{non}-\mathrm{stressed}\,\mathrm{plants}}-1\right)$$

To quantify resilience, we used Equation (6) according to Orwin and Wardle (2004).

(6) $$r=\frac{2\,\left|D_{0}\right|}{(|D_{0}|+\left(|D_{x}|\right)}-1$$

where *D*_0_ is the difference between the biomass of the non-stressed plants and the stressed plants at the end of the drought event at (t_0_) and D_x_ is the difference between the non-stressed plants and the stressed plants at the time point t_x_ chosen to measure resilience (harvests H3, H4, and H5). This resilience index *r* is bounded by −1 and +1, with maximal resilience at +1. This index is standardized by the amount of change initially caused by the drought (D_0_), as this determines the state from which it has to recover.

### Statistical Analysis

In the drought resistance experiment, CCB and WUE were non-normally distributed according to the Shapiro-Wilk test and the variances between the groups were homogeneous according to Levene’s test in the *car* R-package (John and Weisberg, 2011). To satisfy normality criteria a generalized linear model was used with quasipoisson error distribution and a log link function in the *nlme* package (Pinheiro et al., 2018). The model included two independent variables representing *DIV* and *CD*, and their interaction *DIV* × *CD*. Block effect was removed from the model because it did not show improvement in the model according to Akaike’s information criterion (AIC) (Burnham et al., 2011). Following ANOVA, Tukey’s HSD test at α = 0.05 was used to determine the significance of differences among the treatments’ mean values at a given irrigation regime by using the *Agricola* R- package (De Mendiburu, 2019).

In the resilience experiment, CCB, WUE, and CGR at the different harvest times were evaluated with ANOVA. In a first step, a model was used with *DIV*, *DE*, *Har* and all possible interactions between the three factors. As a further step, to provide an easier interpretation to the performance of the treatment independent of the drought stress event, the data of non-stressed plant and stressed plants were analyzed separately as submodels with two factors (*DIV*, *Har*, and *DIV* × *Har*). In each sub-model, the normality of residuals was checked. Block effect was almost significant and showed an improvement in the model according to AIC (Burnham et al., 2011); therefore it was considered in all submodels to create consistent comparison among the models. After ANOVA, Tukey’s HSD test at α = 0.05 was used to determine the significance of differences among the mean values of the treatments at a given harvest time by using the *Agricola* package (De Mendiburu, 2019). The significance of LER above one was tested by using two-sided Welch’s *t*-tests against 1. All statistics were performed using R (version 3.6.1) with R studio (version 1.1.463) (R Core Team, 2019).

## Results

### Diversity and Drought Resistance

**FIGURE 1:**
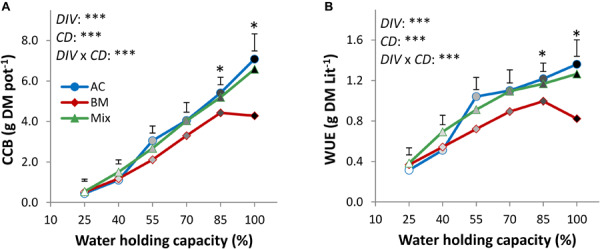
Cover crop aboveground biomass (CCB, dry matter; **A**) and water use efficiency (WUE; **B**) of alsike clover (AC) and black medic (BM) in monocultures and a 1:1 mixture of the two species (Mix) in response to six intensities of cumulative drought (100, 85, 70, 55, 40, and 25% WHC) visualized in black gradient color. Vertical bars represent Tukey’s HSD test (*p* < 0.05) at a given WHC (%) of *n* = 4. Asterisks above the vertical bars indicate significant differences among the mean of the monocultures and the mixture at each drought intensity (WHC; %) based on one-way ANOVA at *P* < 0.05. However, asterisks next to the factors *DIV*, *CD*, and *DIV* × *CD* indicate significant effects based on ANOVA results of the generalized linear model; ^∗∗∗^*P* < 0.001, ^∗^*P* < 0.05.

In the drought resistance experiment, the factors *DIV*, *CD*, and *DIV* × *CD* significantly affected crop biomass. A level of 25% WHC reduced CCB by 93.8, 87.9, and 91.8% in AC_mono_, BM_mono_, and the mixture, respectively, compared to full irrigation (Figure 1A and Supplementary Table S1), thus revealing slightly higher drought resistance in BM_mono_ than in AC_mono_. The AC_mono_ produced more CCB than the other treatments from 55 to 100% WHC, with significantly higher biomass than BM_mono_ by 37.5 and 39.5% at 85 and 100% WHC, respectively. The mixture biomass was not significantly different from AC_mono_ at any drought intensity but was significantly higher than BM_mono_ by 32.7 and 34.9% at 85 and 100% WHC, respectively.

The cumulative drought effect on WUE showed a similar trend as CCB in all treatments, with a reduction by 76.9, 55.3, and 69.6% in AC_mono_, BM_mono_, and the mixture, respectively, at 25% WHC compared to full irrigation (Figure 1B and Supplementary Table S2), again confirming higher drought resistance in BM_mono_ than in AC_mono_. AC_mono_ was higher in WUE than the other treatments at drought intensity from 55 to 100% WHC. Specifically, at 85 and 100% WHC, the WUE of AC_mono_ was significantly higher than BM_mono_ by 85.9 and 88.4%, respectively, but it was not different from that of the mixture.

**FIGURE 2:**
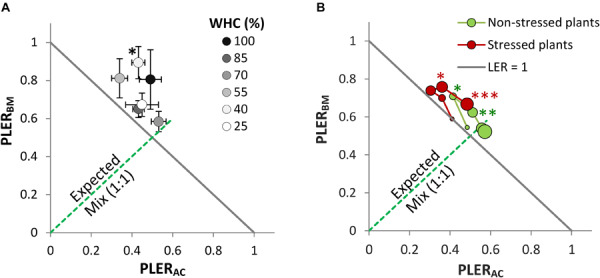
Partial Land equivalent ratio of alsike clover (PLER_AC_) and black medic (PLER_BM_) in a binary mixture of the two species (1:1) in two experiments simulating two drought scenarios: (1) cumulative drought **(A)** with six drought intensities as in Figure 1, and (2) a drought event **(B)** with two treatments of non-stressed plants (green symbols) and the stressed plants (red symbols). Successive harvest times are visualized by increasing symbol size. The solid gray line corresponds to a land equivalent ratio = 1 (LER = PLER_AC_ + PLER_BM_). The broken green line represents the expected PLER for the mixture. Asterisks above some data points represent a significant increase in LER >1 (*P* < 0.05) according to Welch’s *t*-test; ^∗∗∗^*P* < 0.001, ^∗∗^*P* < 0.01, ^∗^*P* < 0.05.

The LER was >1 at the different drought intensities, but this was only significant at 40% WHC (Figure 2A). Notably, there was no consistent directional effect of drought intensity on LER. Drought intensity did not significantly correlate with LER (*r* = 0.29, *P* = 0.57, *df* = 4), nor with the AME (*r* = 0.07, *P* = 0.90, *df* = 4). In the mixture, BM was dominant and at all drought intensities showed PLER_BM_ above 0.5, with a minimum of 0.58 at 70% WHC and a maximum of 0.89 at 40% WHC. Thus, there was also no trend of PLER_BM_ along the drought intensities. Conversely, PLER_AC_ was lower than 0.5 at most of the drought intensities.

### Diversity and Drought Resilience

All mixtures, both when non-stressed and when drought-stressed, showed LER >1 after H2 but this was not consistently significant (Figure 2B). In the non-stressed plants, the increase in LER was significantly larger than one only at H2 and H3 while this was the case in the drought-stressed plants only at H4 and H5. A trend was observed in the PLERs over time independent of the drought event, where BM dominated at the early stages of growth (points moving toward higher PLER_BM_ and lower PLER_AC_ over time) whereas AC gradually contributed relatively more to the mixture at later stages (points moving back toward lower PLER_BM_ and higher PLER_AC_).

**FIGURE 3:**
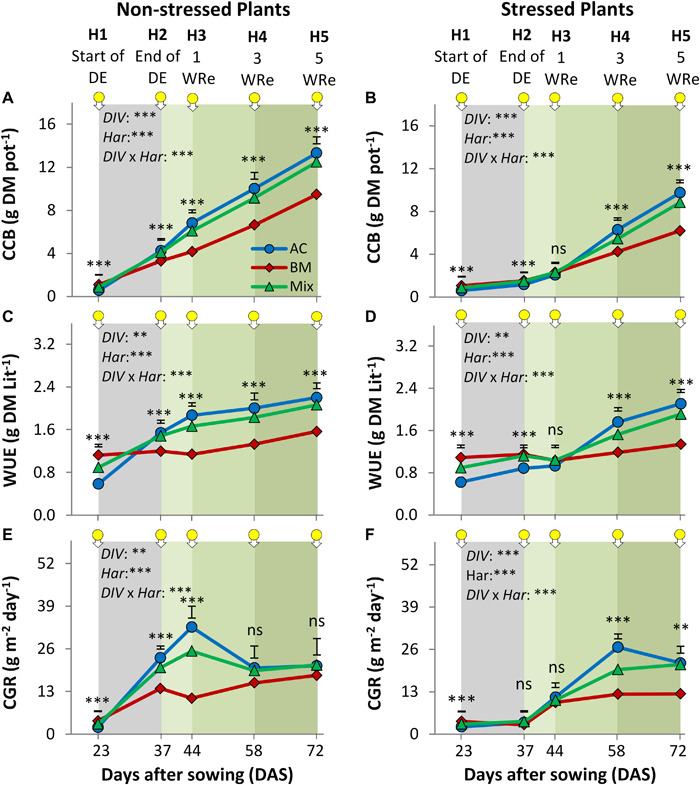
Cover crop aboveground biomass (CCB; **A,B**), water use efficiency (WUE; **C,D**), and crop growth rate (CGR; **E,F**) of alsike clover (AC), black medic (BM) in monocultures and a 1:1 mixture of the two species (Mix). The non-stressed plants **(A,C,E)** and stressed plants **(B,D,F)** were harvested five times indicated by the downward arrows tagged with yellow small circles. The stressed plants were subjected to a drought event (DE; gray shaded) for 14 days. The green gradient color represents weeks of recovery (WRe) after rewatering. Vertical bars represent Tukey’s HSD test (*P* < 0.05) at a given harvest time (*n* = 5). Asterisks above the vertical bars indicate significant differences among the mean of the monocultures and mixture at each harvest time based on one-way ANOVA at *P* < 0.05. However, asterisks next to the factors *DIV*, *Har*, and *DIV* × *Har* indicate significant effects based on ANOVA results of the generalized linear model; ^∗∗∗^*P* < 0.001, ^∗∗^*P* < 0.01, ns, not significant.

In the drought resilience experiment, the factors *DIV*, *Har*, and *DIV* × *Har* significantly affected CCB, WUE and CGR in the non-stressed and drought-stressed plants (Figure 3 and Supplementary Tables S3, S4). In the non-stressed plants from H2-H5, the AC_mono_ produced more CCB than BM_mono_ up to 28.8%, and in the stressed plants from H4-H5 up to 36.2% (Figures 3A,B). The mixture biomass was not significantly different from AC_mono_ at any of the harvest times.

The effect of the DE on WUE at H1 showed a similar trend as CCB in all diversity treatments where AC_mono_ was significantly higher than BM_mono_ at H5 up to 40.8 and 36.4% in the non-stressed plants and drought-stressed plants, respectively (Figures 3C,D).

The dynamics of CGR in the non-stressed plants showed that AC_mono_ reached a peak at H3 which was by 40.1% higher than CGR of BM_mono_, but did not differ from the Mix (Figure 3E). However, in the stressed-plants, AC_mono_ reached a peak of CGR at H4 with significantly higher values than BM_mono_ and the Mixture by 54.0 and 25.6%, respectively (Figure 3F).

**FIGURE 4:**
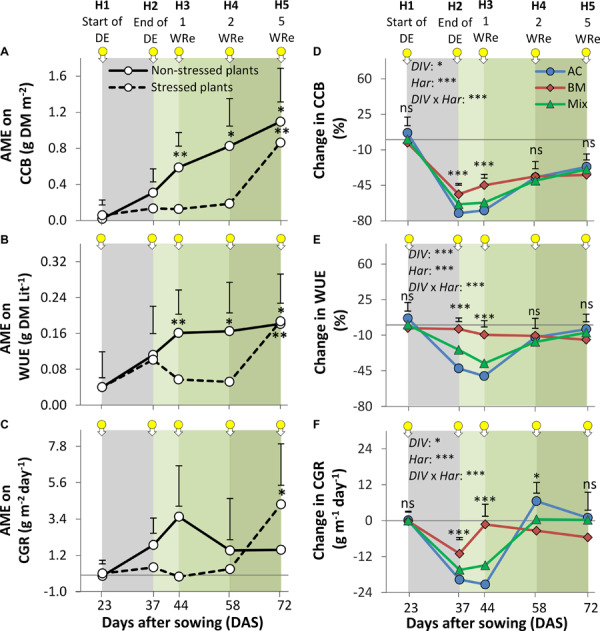
Absolute mixture effect (AME) on cover crop aboveground biomass (CCB; **A**), water use efficiency (WUE; **B**), and crop growth rate (CGR; **C**) of alsike clover (AC), black medic (BM) in monocultures and a 1:1 mixture of the two species (Mix). Mixture effect = the estimated value in mixture – the estimated value in average of monocultures. Positive values indicate positive mixture effect. Change in the CCB **(D)**, WUE **(E)**, and CGR **(F)** (see Equation 5). The plants were harvested at five times indicated by the downward arrows tagged with yellow small circles. The gray shaded area indicates the duration of a drought event and the green gradient color represents weeks of recovery (WRe) after rewatering. In **(A–C)**, the non-stressed plants and stressed plants are visualized in solid and broken lines, respectively, the vertical bars represent LSD test (*p* < 0.05) at a given harvest time (*n* = 5) and the asterisks indicate significant mixture effects above 0 according to Welch’s *t*-test. In **(D–F)** The vertical bars represent Tukey’s HSD test (*p* < 0.05) at a given harvest time (*n* = 5). Asterisks above the vertical bars indicate significant differences among the mean of the monocultures and mixture at each harvest time based on one-way ANOVA at *P* < 0.05. However, asterisks next to the factors *DIV*, *Har*, and *DIV* × *Har* indicate significant effects based on ANOVA results of the generalized linear model; ^∗∗∗^*P* < 0.001, ^∗^*P* < 0.05, ns, not significant.

The absolute mixture effect on CCB, i.e., the difference between the observed value in the mixture and the average value of the sole crops, increased over time and tended to be higher in the non-stressed than in the drought-stressed plants, especially at H3 and H4 (Figure 4A). Similar trends were observed for WUE and CGR (Figures 4B,C).

Further data analysis was performed on the effect of the drought event, measured as the relative difference between drought-stressed and non-stressed plants to understand the dynamics of biomass, WUE, and CGR. Shortly after the event, drought affected all three variables less in BM_mono_ than in AC_mono_, with the mixture showing intermediate values (Figures 4D–F). With regard to CGR, BM_mono_ recovered faster than AC_mono_, with the mixture again showing intermediate behavior (Figure 4F). At the final harvest, however, differences in the drought effect on CGR were not significant among treatments.

**FIGURE 5:**
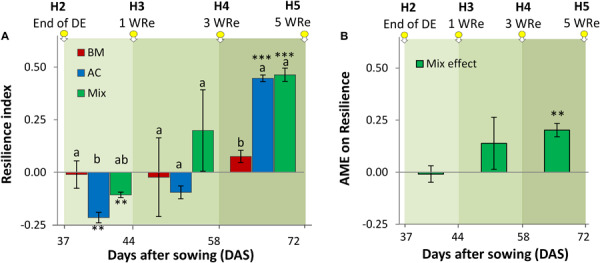
Resilience index calculated to alsike clover (AC), black medic (BM), and a 1:1 mixture of the two species (Mix) after recovery from a drought event **(A)** and absolute mixture effect on resilience (AME; resilience in mixture – average resilience in the monocultures) **(B)**. The three harvest times during the recovery time are indicated by the downward arrows tagged with yellow small circles. The green gradient color represents weeks of recovery (WRe) after rewatering. The vertical bars represent the standard error. Different letters above the vertical bars indicate significant differences among the three treatments at a given harvest based on ANOVA followed by Tukey’s HSD test (*p* < 0.05). Asterisks indicate significant resilience in comparison with 0 according to Welch’s *t*-test at *p* < 0.05.

The resilience index, quantifying the capacity of biomass to recover after the drought event, increased over time and showed a significant diversity treatment effect, which varied over time (Figure 5A). At H3, 1 week after cessation of the drought event, recovery was negative in sole AC and in the mixture, but not different from zero in BM_mono_. While at H4 no significant differences were observed in the recovery among the diversity treatments, a significantly higher resilience index was found for AC than for BM at H5. The absolute mixture effect of the resilience index increased over time and was significantly positive at the last harvest, i.e., the mixture recovered more fully than expected from the average recovery of the two-component monocultures (Figure 5B).

## Discussion

### Effects of Species Identity and Mixing on Drought Resistance

Our drought resistance experiment confirmed previous research (Döring et al., 2013; Storkey et al., 2015; Elsalahy et al., 2019; Komainda et al., 2019) that BM is more drought-resistant than AC. Relative to the maximum biomass potential, which was higher in AC than in BM, the biomass under constant drought conditions was similar in both species, so that the relative biomass-reducing effect of drought was stronger in AC than in BM (Figure 1A). However, differences between the species were smaller than expected – in fact, both species had a relatively strong capacity to tolerate drought, possibly because the slowly imposed constant drought triggered drought stress memory in the plants to adjust structure, metabolism, and function to withstand drought (Fleta-Soriano and Munné-Bosch, 2016). This would explain why AC was able to perform similarly to BM under extreme drought (Figure 1A) despite AC’s reported drought sensitivity (Chapman et al., 1990; Sheaffer et al., 2003). Under conditions of 100% WHC, AC produced more biomass than BM, which was anticipated as AC is known to be well-adapted to wet conditions (Döring et al., 2013). BM, on the other hand, was negatively affected at 100% WHC, confirming that BM grows better in well-drained soil while insufficient oxygen in water-saturated soil impairs its growth (Döring et al., 2013; Elsalahy et al., 2019). Further measurements on root biomass showed no significant difference between the two species. However, assessing the root:shoot ratio of both species showed potentially different plant-specific strategies of resource uptake in response to drought. Remarkably, BM showed no significant difference in root:shoot ratio at the different cumulative drought intensities, suggesting that BM may resist drought without altering root:shoot allocation. However, the root:shoot ratio of AC showed a significant difference by responding to drought intensity, with large differences between the well-watered plants at 100% WHC and the extreme drought treatment at 25% WHC. This observation suggests that AC as a slow-growing species may exhibit high phenotypic plasticity against cumulative drought (Supplementary Figure S4). More importantly, we did not find any consistent mixture effect on drought resistance, in line with previous research (Van Ruijven and Berendse, 2010; Lanta et al., 2012; Grossiord et al., 2014). Although WUE in the mixture was increased in comparison with the average WUE of the monocultures (Figure 1B), confirming earlier results (Mariotte et al., 2013), the relative advantage of the mixture over the monocultures was not affected by drought intensity (Figure 2A). We see two potential reasons for this lack of association between drought intensity and mixture effect.

First, mixture effects are expected to be driven by reduced competition among individual plants, so that total resource use is greater in the mixture than in the monocultures (Bedoussac et al., 2015). In our case, however, competition between plants may just have been relatively independent of water availability, so that any benefits of reduced competition in the mixture could not play out differentially across the levels of drought intensity. Under conditions of constant low water availability, plant growth was restricted and therefore the zone of influence of these plants would have been small (Schwinning and Weiner, 1998), whereas full irrigation led to expanded plant size and thereby to an increased zone of influence. Thus, under constant drought the effects of reduced resource availability (leading to increased competition among plants) and reduced plant size (leading to decreased competition) may partly have canceled out, so that any competition related effects of mixing would be little affected by drought intensity.

Second, complementarity for water use may be possible through different spatiotemporal growth patterns of the two species, i.e., asynchronous root development, but as we harvested the plants only once we potentially missed the dynamic interaction between the plants, in particular any asynchrony. Also, spatial complementarity may be limited in a pot experiment where root space is restricted, though such restriction may also be partly the case under field conditions, e.g., due to soil compaction.

### Effects of Species Identity and Mixing on Drought Resilience

In line with previous findings (Van Peer et al., 2001; Allsion, 2004; Di Falco and Chavas, 2008; Hutchison et al., 2018), we found that mixing species promoted resilience (Figure 5B). Notably, however, we also found that resilience to drought is possible with only one species present (Figure 5A), i.e., without any response diversity. This is in contrast to previous statements emphasizing that resilience requires response diversity (Tilman and Downing, 1994; Mori et al., 2013). Yet from an evolutionary perspective, individual plants will benefit from the ability to recover after a drought event, so any physiological mechanism that allows faster or fuller recovery will be selected for. It is therefore expected that even in a plant monoculture, resilience to drought will occur. The basis for recovery will be reserves built up during the pre-stress period, e.g., in the roots. In addition, an indirect mechanism may lead to a high resilience index in both single species and mixed species stands. During the drought event, stressed plants will be reduced in their growth in comparison to the non-stressed plants. Therefore, after cessation of the drought more resources (e.g., nutrients) will remain for the stressed than for the non-stressed plants. In effect, this leads to an apparent “recovery” of the stressed plants.

Further, we observed a strong effect of species identity on drought resilience, with a fuller recovery in AC than in BM (Figure 5A). The two species showed asynchronous behavior, as BM recovered faster, while AC recovered later (Figure 4F). This also means that different aspects of resilience may trade off: recovery tended to be faster in BM, but was achieved to a fuller degree in AC. Our observations confirm BM as a relative fast-growing species (Döring et al., 2013; Elsalahy et al., 2019; FAO, 2019), and AC as growing more slowly (Elsalahy et al., 2019). While fast-growing species do not build large reserves for later growth (Diaz and Cabido, 2001; Hoover et al., 2014), slow-growing species have low demands at the early stages and therefore are less likely to exhaust limiting resources (Poorter, 1989). The fact that AC_mono_ recovered more fully from the drought event than BM (Figure 5A) may also be explained by the difference in growth dynamics, as the slow-growing AC may have built more reserves for later growth (Poorter, 1989). In the drought resilience experiment, AC_mono_ was more affected by the drought than BM_mono_ shortly after the drought event, but later AC_mono_ became resilient via a strategy of slow recovery that contributed to significantly larger CCB, WUE, and CGR than observed in BM (Figures 4D–F). This is in contrast to the finding that slow species tend to have high resistance but low resilience as they recover slowly and therefore, cannot restore productivity (Diaz and Cabido, 2001; Oliver et al., 2015; Hofer et al., 2016; Craven et al., 2018), while mixtures dominated by fast-growing species tend to have high recovery and resilience but low resistance (Diaz and Cabido, 2001; Hoover et al., 2014; Craven et al., 2018).

The combination of fast-slow dynamics may stabilize biomass production in response to drought as it generates differences in the peak biomass at different dates (Loreau and de Mazancourt, 2013) and may play a role in drought resilience. The fast-growing species use an exploitative strategy to acquire resources and therefore govern faster recovery, while the slow-growing species use a conservative strategy to tolerate stress which may promote resistance but with lower capacity to recover (Oliver et al., 2015). The observed asynchrony between AC and BM may have contributed to the diversity-effect on resilience, as in the mixture asynchronous growth will lead to temporal niche differentiation and reduced interspecific competition, thereby increasing overall productivity. Accordingly, fast-slow functional diversity may be adaptive to environmental perturbations (Bybee-Finley et al., 2016), specifically in equiproportional mixtures (Kirwan et al., 2007).

### Transferability of Results

The results reported in the current study for plants grown in pot experiments, although artificial, gave similar qualitative (direction of the response) and quantitative (absolute biomass) estimates of AC and BM grown in the field (Supplementary Figure S5). In a field experiment on mixing AC and BM (Elsalahy et al., 2019), the experimental conditions were close to the drought resistance experiment at 70% WHC. In both the field experiment and the greenhouse experiments reported here, the plants were sown in the same year, at the same seed density, soil type, moisture level, and harvested at a similar phenological stage. During the growing season of the field experiment, the plants received ∼70% precipitation of the long-term average (1981–2010) and produced biomass comparable to that at drought intensity 70% WHC in the pot experiment, suggesting that results of the pot experiments are comparable to field conditions when assessing short-term drought effects on these legume species.

Nevertheless, it is clear that our pot experiments are unable to replicate or represent the complex drought effects on crop yields in agro-ecosystems that are generated by the various interactive drought-related mechanisms, involving the soil as well as biotic factors (Mariotte et al., 2018; Schimel, 2018).

Further, we only used two species in this study, in order to restrict the complexity of the potential interactions and we only varied species richness but not any other aspects of diversity. Therefore, our conclusions on the effect of diversity on resilience and resistance of plants remain limited to this relatively simple set-up. However, the fact that we observed significant resilience to drought in a single-species stand is unaffected by this limitation. Further studies will need to clarify how individual species contribute to resilience in more complex communities, and how large these contributions are in relation to the effect of diversity.

Finally, we acknowledge that the positive effects of species richness under dry conditions, for which our study shows some evidence, will not be sufficient for reducing the large negative impacts of drought on crops.

## Conclusion

Our main aim was to quantify the effects of mixing plant species on resistance and resilience to drought. Representing an arable context of a short-term green manure, we chose a basic design, by mixing two species belonging to the same functional group, legumes, and comparing the mixture with the two monocultures of the component species, as is common in intercropping research (Bedoussac et al., 2015). The two legume species differed in their drought resistance and resilience, with BM being more resistant than AC, while the order was reversed for resilience. Mixing the two species was not more advantageous under constant drought than under fully irrigated conditions, suggesting that species identity was more important than species richness in response to constant drought. However, the mixture was more resilient to a transient drought event than the average of the sole crops. Remarkably, a monoculture of AC was equally resilient to drought as the mixture, indicating that response diversity was not required for the plants to show resilience.

The strong species identity effects on drought resilience in our study suggest that further research is needed to determine drought resistance and resilience in a larger range of (agriculturally relevant) plant species, including those grown in arable systems. Also, with the positive effect of mixing species on drought resilience shown in our experiments, there is great potential to apply these findings to other relatively simple (e.g., binary) plant mixtures, possibly adding to the already numerous benefits these mixtures offer for agriculture. Further studies on the physiological mechanisms of resilience to drought in plants will also help to deepen the understanding of the underlying processes.

## Data Availability Statement

All datasets generated for this study are included in the article/Supplementary Material.

## Author Contributions

HE and TD conceived and designed the experiments, conducted the experiments, collected the data, wrote the manuscript, and performed the statistical tests. TD provided the facilities and advised on the preparation of materials. SB-K, TK, and C-LR read and edited the manuscript. All authors approved the final manuscript.

## Conflict of Interest

The authors declare that the research was conducted in the absence of any commercial or financial relationships that could be construed as a potential conflict of interest.
