# Supplementary material for: Crop Resilience to Drought With and Without Response Diversity
Source: Front Plant Sci. 2020 Jun 3;11:721. doi: 10.3389/fpls.2020.00721 (PMC7283915; doi:10.3389/fpls.2020.00721)
Supplement: Supplementary file 1 [file Data_Sheet_1.pdf]

Supplementary Material

Soil, climatic variability, and experimental considerations

In both pot experiments, under optimal water conditions (95% - 100% WHC), the difference in soil fertility and the fluctuation in the daily temperature did not significantly affect the overall performance of AC and BM. To compare plants at the same growth stage, the growing degree days (GDD; °Cd) of BM was calculated by using a base temperature of 0.6 °C (Tribouillois et al., 2016). The GDD, defined as the sum of degrees by which each day's mean air temperature was higher than the base temperature, was calculated from the date of sowing until the date of the sampling. Based on the GDD, the BM was harvested in the drought resistance experiment at 986 °Cd (50 DAS), while in the resilience experiment at H4, at 1070 °Cd (58 DAS). These harvest times were at the same phenological stage (10%-30% flowering) but the crop biomass was slightly lower in the drought resistance experiment than the resilience experiment by -13.1%, -9.8%, and -21.2% of AC, BM, and Mix, respectively.

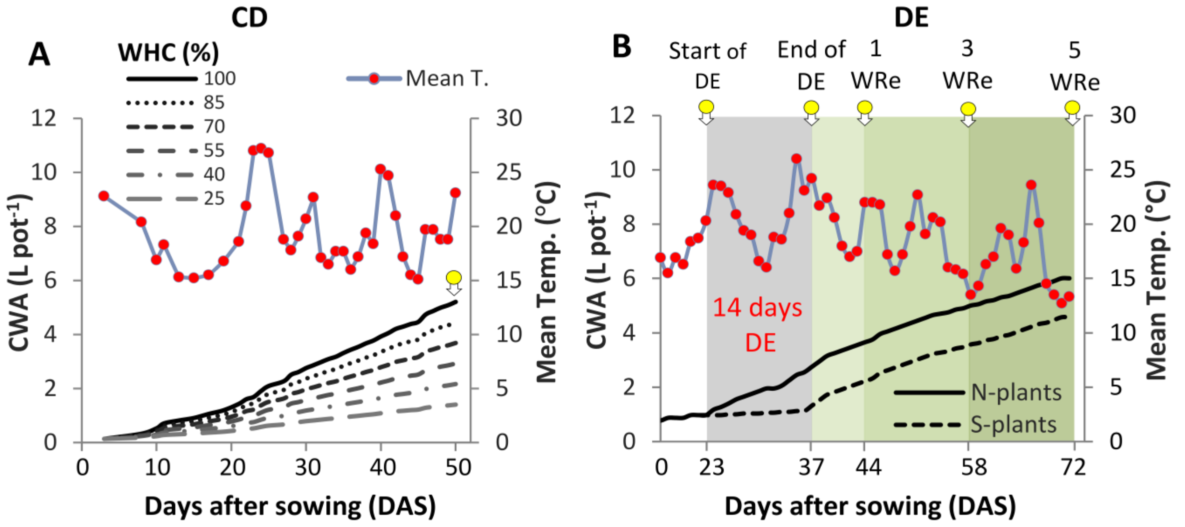

FIGURE S1 | Cumulative water applied per pot (CWA) and the mean daily temperature (Mean Temp.) in two experiments simulating two drought scenarios: (1) cumulative drought (CD; Panel A) with six drought intensities 100%, 85%, 70%, 55%, 40%, and 25% WHC and (2) a transient drought event (DE; grey shaded; panel B) with two treatments of non-stressed plants (solid line) and the stressed plants (broken line). The downward arrows tagged with yellow small circles indicate the harvest times in each experiment. The green gradient color represents weeks of recovery (WRe) after rewatering (1WRe; light green, 3WRe; medium green shaded and 5WRe; dark green shaded).

Tribouillois, H., Dürr, C., Demilly, D., Wagner, M. H., and Justes, E. (2016). Determination of germination response to temperature and water potential for a wide range of cover crop species and related functional groups. *PLoS One* 11, 1–16. [Cross Ref].

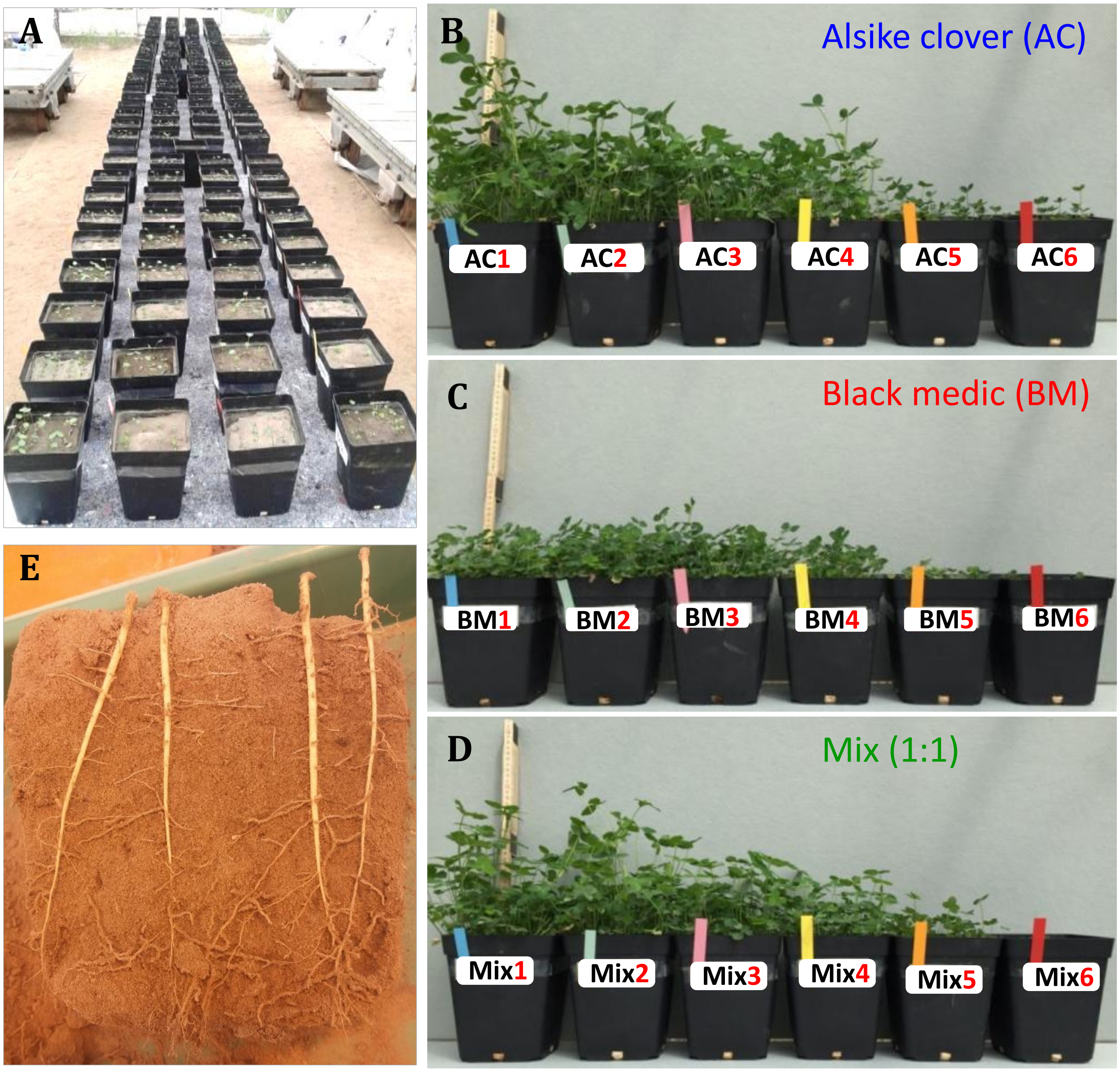

FIGURE S2 | Drought resistance experiment conducted on alsike clover (AC) and black medic (BM) in monocultures and a 1:1 mixture of the two species (Mix) in response to six intensities of cumulative drought (CD; 100%, 85%, 70%, 55%, 40%, and 25% WHC). A: The experimental setup with 4 blocks each contains 18 pots. One block is represented in B: The cover crop aboveground biomass (CCB) of AC, C: The CCB of BM, and D: The CCB of the Mix. E: A side view of the roots of some individual plants of BM at 100% WHC.

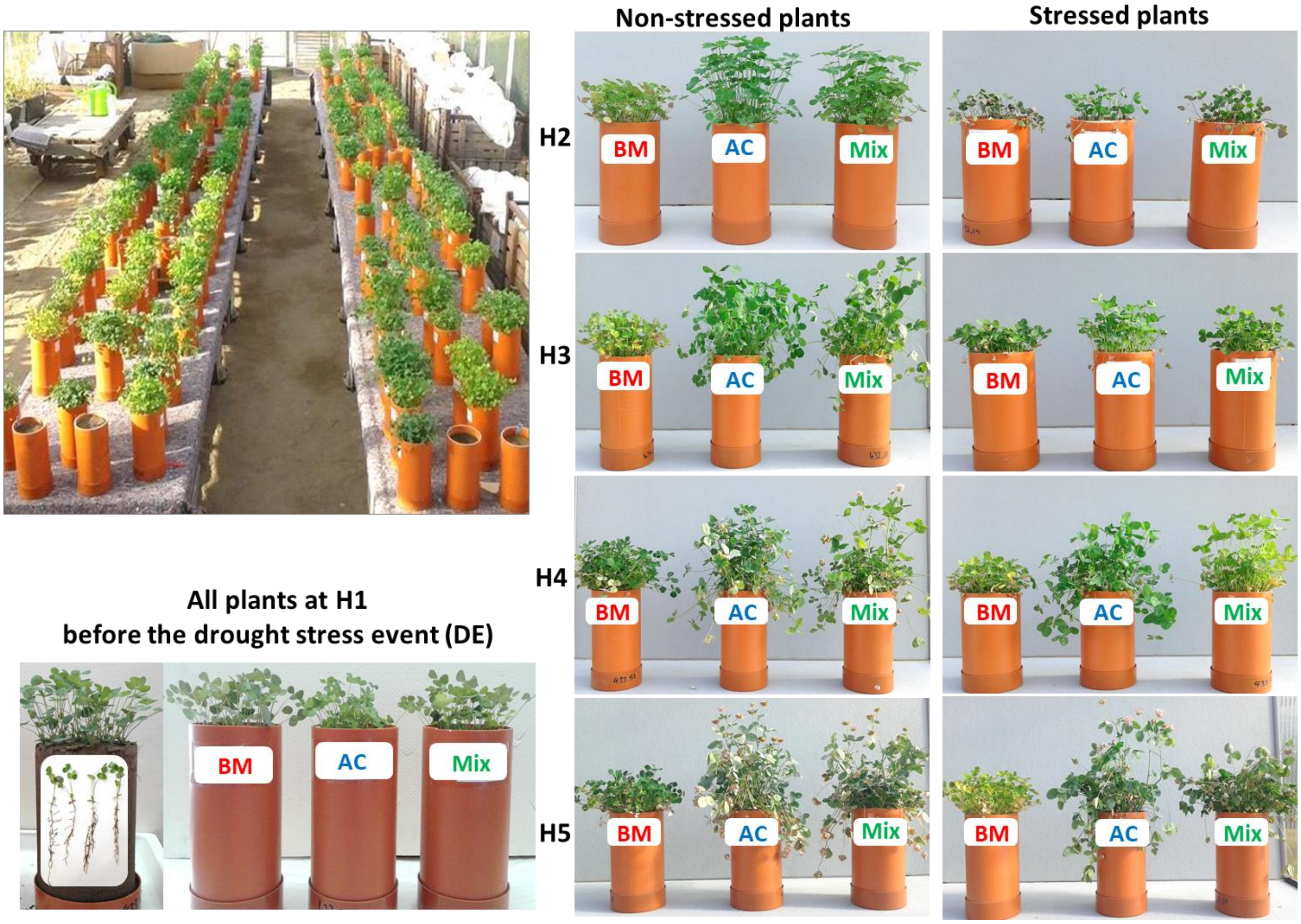

FIGURE S3 | Drought resilience experiment conducted on alsike clover (AC) and black medic (BM) in monocultures and a 1:1 mixture of the two species (Mix) in response to a transient drought event (DE) with two treatments of non-stressed plants and the stressed plants. The plants were harvest five harvest times: H1; 23, H3; 37, H3; 44, H4; 58, and H5; 72 days after sowing.

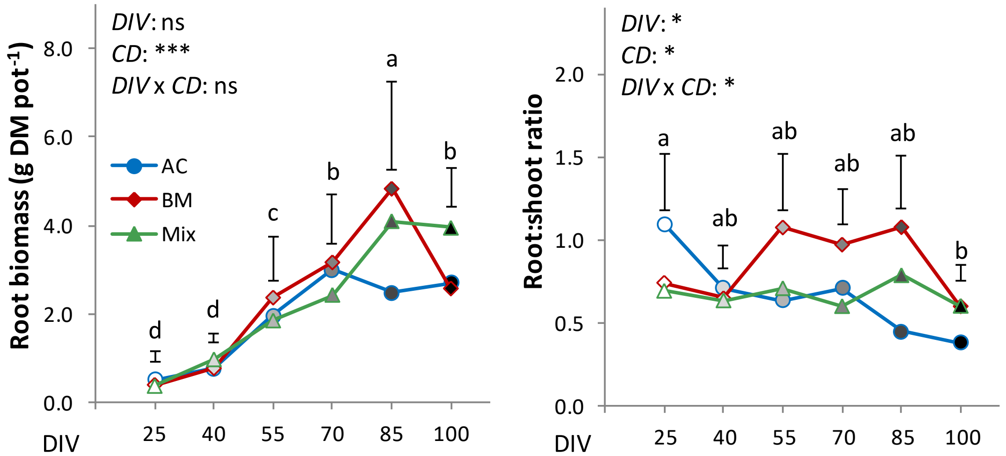

DIV	25	40	55	70	85	100
DF	2,9	2,9	2,9	2,9	2,9	2,9
F-value	0.44	1.55	0.31	0.47	1.96	2.91
p-value	0.66	0.39	0.74	0.64	0.20	0.11
AC	a	a	a	a	a	a
BM	a	a	a	a	a	a
Mix	a	a	a	a	a	a

Water holding capacity (%)

DIV	25	40	55	70	85	100	CD		
							DF	F-value	p-value
DF	2,9	2,9	2,9	2,9	2,9	2,9			
F-value	1.60	0.29	1.79	3.05	3.98	4.15			
p-value	0.25	0.75	0.22	0.1•	0.06•	0.05•			
AC	a	a	a	a	b	a	5,15	5.44	0.005**
BM	a	a	a	a	a	a	5,15	2.49	0.08
Mix	a	a	a	a	ab	a	5,15	0.49	0.78

Water holding capacity (%)

FIGURE S4 | Root biomass (DM; dry matter per pot) and root:shoot ratio of three diversity treatments (DIV) including alsike clover (AC) and black medic (BM) in monocultures and a 1:1 mixture of the two species (Mix) in response to six intensities of cumulative drought (CD; 100%, 85%, 70%, 55%, 40%, and 25% WHC) visualized in black gradient color. Vertical bars represent Tukey's HSD test ($p < 0.05$) at a given WHC (%) of $n = 4$. Different letters above the vertical bars indicate significant differences among the different intensities of cumulative drought, however, different letters below the figures indicate significant differences among the mixture and the two monocultures, based on ANOVA followed by Tukey's HSD test ($p < 0.05$). Asterisks next to the factors DIV, CD, and DIV x CD indicate significant effects based on ANOVA results of the generalized linear model; *** = $P < 0.001$, * = $P < 0.05$, • = $P < 0.1$, and ns = not significant.

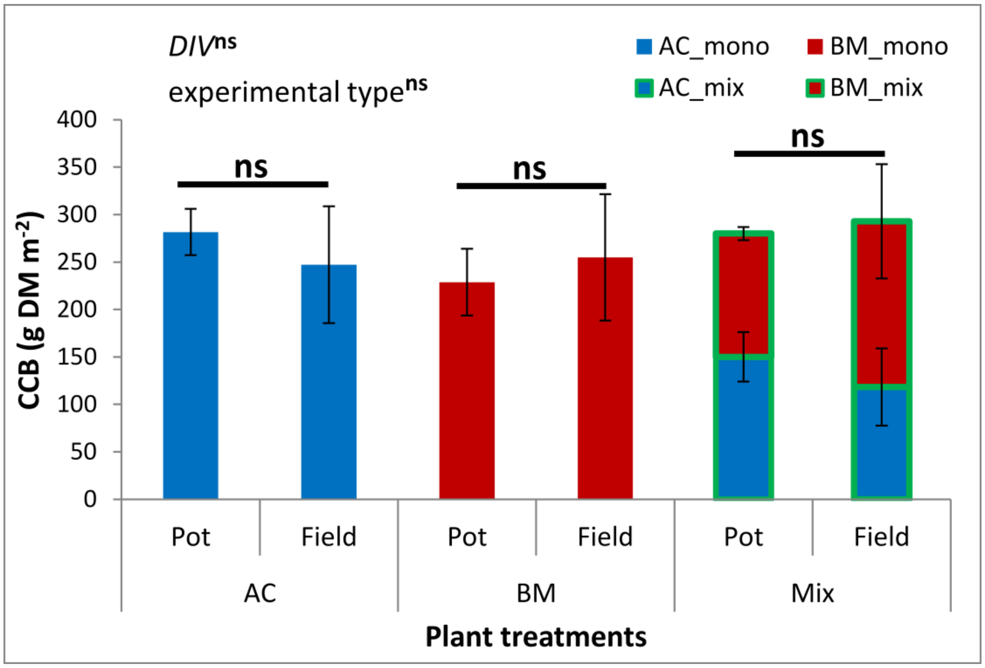

FIGURE S5 | Cover crop aboveground biomass (CCB) of Alsike clover (AC) and Black medic (BM) in monocultures and a 1:1 mixture of the two species (Mix) in two experiments with different experimental types (Pot vs Field). In both experiments, the plants were sown at the same seed density, grown at the same soil type, moisturized at almost the same level, and harvested at the same growing degree days (GDD; °Cd). In the pot experiment, the moisture level represented 70% of the maximum water-holding capacity and in the field experiment (Field FU9_2016; Elsalahy et al., 2019) the plants received during the growing season 70% precipitation than the long-term average from 1981-2010. The plants were harvested in the pot experiment at 50 days after sowing (DAS) at 986 °Cd and in the field experiment after 64 DAS at 1100 °Cd. Vertical bars represent the standard deviation of the means of n = 4 and n = 12 in the pot and field experiments, respectively. Statistics above the horizontal bars represent the non-significant difference between the same treatments at the two soils according to Welch's t-test at $P < 0.05$; ns = not significant.

Elsalahy, H., Döring, T., Bellingrath-Kimura, S., and Arends, D. (2019). Weed Suppression in Only-Legume Cover Crop Mixtures. *Agronomy* 9, 648. [Cross Ref].

TABLE S1 | Summary of the generalized linear model (GLM) describing the response variables cover crop aboveground biomass (CCB), water use efficiency (WUE), root biomass, and root:shoot ratio of alsike clover and black medic in monoculture and a 1:1 mixture of the two species. The explanatory variables were plant treatments (DIV; three levels), cumulative drought (CD; six intensities of cumulative drought representing 100%, 85%, 70%, 55%, 40%, and 25% of WHC), DIV x CD, and block as an additive variable. F and P values refer to ANOVA tests of each factor (N = 72).

	Response variables											
Explanatory variables	CCB (g DM pot^{-1})			WUE (g DM Lit^{-1})			Root biomass (g DM pot^{-1})			Root:shoot ratio		
	DF	F-value	P-value	DF	F-value	P-value	DF	F-value	P-value	DF	F-value	P-value
DIV	2,51	31.4	0.00	2,51	28.4	0.00	2,51	1.8	0.18	2,51	4.8	0.01
CD	5,51	302.3	<.0001	5,51	112.4	<.0001	5,51	26.9	0.00	5,51	2.8	0.03
DIV x CD	10,51	6.8	0.00	10,51	5.0	0.00	10,51	2.0	0.06	10,51	2.7	0.01
Block	3,51	4.2	0.01	3,51	4.8	0.01	3,51	6.3	0.00	3,51	5.6	0.00

TABLE S2 | Tukey's HSD test ($p < 0.05$) describes the difference in cover crop aboveground biomass (CCB) and water use efficiency (WUE) of the monoculture of alsike clover (AC) and black medic (BM) and a 1:1 mixture of the two species (Mix) in response to six intensities of cumulative drought (100%, 85%, 70%, 55%, 40%, and 25% WHC) (n = 24).

CCB (g DM pot^{-1})							WUE (g DM Lit^{-1})						
CD (WHC; %)							CD (WHC; %)						
	25	40	55	70	85	100		25	40	55	70	85	100
DF	2,9	2,9	2,9	2,9	2,9	2,9	DF	2,9	2,9	2,9	2,9	2,9	2,9
F-value	1.02	4.18	7.08	3.28	7.66	12.21	F-value	1.02	4.18	7.08	3.28	7.66	12.21
p-value	0.40	0.05	0.01	0.09	0.01	0.003	p-value	0.40	0.05	0.01	0.09	0.01	0.003
AC	a	a	a	a	a	a	AC	a	a	a	a	a	a
BM	a	a	a	ab	b	b	BM	a	a	a	ab	b	b
Mix	a	a	a	b	a	a	Mix	a	a	a	b	a	a

TABLE S3 | Summary of the generalized linear model (GLM) describing the response variables cover crop aboveground biomass (CCB), water use efficiency (WUE), and crop growth rate (CGR) of alsike clover and black medic in monoculture and a 1:1 mixture of the two species. The explanatory variables were plant treatments (DIV; three levels), transient drought event (DE; two levels of non-stressed and stressed plants), Harvest (Har; five harvest times at 23, 37, 44, 58, and 72 days after sowing), DIV x DE , DIV x Har , DE x Har , DIV x DE x Har, and block as an additive variable. F and P values refer to ANOVA tests of each factor (N=380).

	Response variables								
Explanatory variables	CCB (g DM pot^{-1})			WUE (g DM Lit^{-1})			CGR (g m^{-2} day^{-1})		
	DF	F-value	P-value	DF	F-value	P-value	DF	F-value	P-value
Plant treatment (DIV)	2,147	223.0	<.0001	2,147	86.5	<.0001	2,146	58.1	<.0001
Drought event (DE)	1,146	1736.4	<.0001	1,146	240.2	<.0001	1,145	160.1	<.0001
Harvest (Har)	4,142	2958.8	<.0001	4,142	423.6	<.0001	4,141	224.4	<.0001
DIV:DE	2,136	3.7	0.00	2,136	15.6	0.00	2,135	0.4	0.69
DIV:Har	8,128	19.2	0.03	8,128	57.6	0.00	8,127	6.3	0.00
DE:Har	4,124	106.9	<.0001	4,124	35.8	<.0001	4,123	69.4	<.0001
DIV:DE:Har	8,116	8.7	<.0001	8,116	12.0	<.0001	8,115	6.9	0.00
block	4,138	5.5	0.00	4,138	4.3	0.00	4,137	1.1	0.37

TABLE S4 | Tukey's HSD test (p < 0.05) describes the different temporal response of the monoculture of alsike clover (AC) and black medic (BM) and a 1:1 mixture of the two species (Mix) across five harvest times based on the significant interaction of the factors DIV x Har in Figure 3 and Table S2. Harvest times were 23, 37, 44, 58, and 72 days after sowing (n= 5). The response variables are cover crop biomass (CCB), water use efficiency (WUE), and crop growth rate (CGR) of the non-stressed plants, stressed plants, and the percent of change in these variables (n= 5).

Non-stressed plants

DIV	DF	F-value	p-value	DAS 23	DAS 37	DAS 44	DAS 58	DAS 72
CCB (g DM Pot^{-1})								
AC***	4,20	952	<2e-16	e	d	c	b	a
BM***	4,20	57	1.4E-10	d	c	c	b	a
Mix***	4,20	628	<2e-16	e	d	c	b	a
WUE (g DM Lit^{-1})								
AC***	4,20	277	<2e-16	d	c	b	b	a
BM**	4,20	5	5E-03	b	b	b	ab	a
Mix***	4,20	116	2E-13	d	c	b	b	a
CGR (g m^{-2} day^{-1})								
AC***	4,20	44	1E-09	c	b	a	b	b
BM*	4,20	3	3E-02	b	ab	ab	ab	a
Mix***	4,20	44	1E-09	c	ab	a	b	ab

Stressed plants

DIV	DF	F-value	p-value	DAS 23	DAS 37	DAS 44	DAS 58	DAS 72
CCB (g DM Pot^{-1})								
AC***	4,20	2249	<2e-16	e	d	c	b	a
BM***	4,20	276	<2e-16	d	d	c	b	a
Mix***	4,20	878	<2e-16	e	d	c	b	a
WUE (g DM Lit^{-1})								
AC***	4,20	600	<2e-16	d	c	c	b	a
BM***	4,20	8	0.001	b	b	b	ab	a
Mix***	4,20	154	1E-14	d	c	cd	b	a
CGR (g m^{-2} day^{-1})								
AC***	4,20	260	<2e-16	d	d	c	a	b
BM***	4,20	17	3E-06	b	b	a	a	a
Mix***	4,20	98	8E-13	c	c	b	a	a

Change in

DIV	DF	F-value	p-value	DAS 23	DAS 37	DAS 44	DAS 58	DAS 72
CCB (%)								
AC***	4,20	243	<2e-16	a	d	d	c	b
BM***	4,20	14	1.6E-05	a	b	b	b	b
Mix***	4,20	158	8E-15	a	d	d	c	b
Change in WUE (%)								
AC***	4,20	107	3.3E-13	a	c	c	b	b
BM	4,20	0	9.0E-01	a	a	a	a	a
Mix***	4,20	40	3E-09	a	c	d	bc	b
Change in CGR (g m^{-2} day^{-1})								
AC***	4,20	76	8E-12	b	c	c	a	ab
BM	4,20	1364	3.2E-01	a	a	a	a	a
Mix***	4,20	49	5E-10	a	b	b	a	a
